# Affections of the Teeth

**Published:** 1893-09

**Authors:** C. N. Peirce


### International Dental Journal.

Vol. XIV. September, 1893. No. 9.

### Original Communications.[1]

### AFFECTIONS OF THE TEETH.

BY C. N. PEIRCE, D.D.S.

The study of the diseases of the teeth is not of recent origin, but, though of great antiquity, there is still much that remains unwritten which may be suggestive, if not in itself of great value.

The history of dental anomalies and their treatment is almost as old as the history of medicine, and, like the latter, has had its periods of superstition and of mysticism.

To-day, with all the boasted progress dentistry has made in common with other specialties in the healing art, it is in its therapeutic literature, with the exception of publications on bacteriology, but little more than an enumeration, a record, of anomalies and methods of treatment. How and whence these anomalies arose are questions which receive scarcely a respectful consideration. Dental meetings are held and, at these, well prepared papers are read and discussed, but devotion to certain morbid and pathological conditions, with suggestion of remedies, treatment, and manipulative processes, is largely the limitation. This is all, doubtless, very profitable, but does it occur to the reader that it has but little interest or significance save to the specialist?

A new method of treating an abscess, cleansing, enlarging, and

[1] The editor and publishers are not responsible for the views of authors of papers published in this department, nor for any claim to novelty, or otherwise, that may be made by them. No papers will be received for this department that have appeared in any other journal published in the country.

filling a root-canal or a crown cavity, describing the location and the progress of decay and manner of arresting it, inserting a crown or a complete denture, may all be financially successful, but is this narration, even though the methods be novel, the most that can be done towards a contribution to the science of dentistry or to dental therapeutics?

Nature is rich in anomalies, both the flora and fauna are giving countless examples, but does the scientist, who is ever watchful of these, rest satisfied with a record of what he sees? Does he not rather endeavor to step behind the curtain to look for the cause? The phenomenon is, or should be, but preliminary to greater efforts and profounder thoughts. The numerous pathological conditions which affect the teeth in different and diverse individuals make them objects of deep interest and solicitation, but the remarkable, the universal similarity which is recognized in these abnormalities, in their appearance, their progress and termination, renders their appropriate and comparatively successful treatment quite possible within the range of limited skill and mediocre professional attainments. It is unfortunate that the majority of the dental profession are interested only in these anomalous conditions and routine treatment, which is soon fixed within a very limited area, and necessarily so, because the recorded phenomena are, as just stated, so marvellously similar. The inquiry it is desired to stimulate in this paper is, How or whence do these arise, and why so similar in their appearance, frequency, and influence? We are all familiar with the fact that anomalies may make their appearance in the organism of the body without being pathological in effect,—for an example, the absence of a tooth or the reverse, a tooth in addition to the normal number; though disease itself is always an anomaly.

Variations in structure, which come in response to physiological conditions or changes, and also in adaptation to environment, are often in the line of higher development, but are in no sense pathological. It is only when the anomaly impairs the organ's or organism's functions, deteriorates and debases its vigor and shortens its life, that it can be regarded as disease. Symptoms or structural vices are frequently recognized; these are without hesitation said to be the effects, the results, or signs of a series of reactions, and constitute the process. The nearer we can trace this to its inception, the nearer we come to the primary cause or causes, and to the essence of the disease itself.

"Every change recognized in living structures, whether normal or abnormal, is the resultant of forces, incident and inherent, forces from outside as well as from within." "If these are so adjusted or co-ordinated as to preserve the integrity of the part, the result is health, and if sustained not only to the extent of overcoming waste, but in excess of the demand, then growth and development are promoted." "It is this capacity of the organism to appropriate power from external sources that gives life its most distinctive characteristics." When through deteriorated vigor the organism loses this ability, and the inherent power of co-ordination and adaptation is unable to withstand the external disturbing forces, disintegration, disease, and death result. "While integration or growth is the normal end and purpose of vital action, it does not necessarily follow that disintegration or atrophy is abnormal and morbid."

The conflict of forces is never ceasing, there is never equilibrium, never rest; the only living existence of which we are cognizant is in a perpetual struggle. The greater activity of the nutritive processes is in the young and growing being, while as maturity or old age are reached there is a gradual decline, "disintegration being always conditioned by the laws governing integration." "Disease is not an entity, its exciting, its active motive may come from some entity without, but the cause which is most essential and for which we should seek is inherent."

The destroyer whom we would pursue is not an external agent which can be seen and handled, but an element of disorder in the organism itself. The inherent susceptibility of the tissues as a factor in disease is one to which little attention is given in this age of microscopic investigation, the parasitic germ being now held to be the great criminal, the all-important agent; and in consequence of this the part played by a defective organ or organism is amazingly overlooked. All the blame is cast on this poor little bacillus, while the lurking vice in the tissues goes without notice. That the bacillus tuberculosis is the exciting cause in phthisis seems to be well established, but that it may be and is taken into thousands of lungs with perfect impunity is also a well-recognized fact. Before this micro-organism can locate and cause suppurative processes there must be, by acquirement or hereditary predisposition, a soil favorable to its pabulum and habitat.

Dr. Edward Bennett Bronson, to whose article on the origin of types in disease the writer is indebted for much in this paper, says, "The inherent susceptibility of the tissues is a factor in disease to which insufficient attention is given. Every disease rests on a tripod of factors,—to wit, first the primary injury representing the incident force; second, the susceptibility of the part, which stands for the inherent and resisting force; and third, the pathological effect, which is the resultant. To ignore any one of these factors leaves a very unstable basis for pathological study, and however true may be the conclusions arrived at, it stops short of the whole truth."

The same author draws the following conclusion, and the reader will see that it is just as applicable to the dental as to other tissues. Susceptibilities to disease having their origin in defects of development, and seldom peculiar to the individual, but occurring in greater or less degree in the same form throughout a large portion of the human family, are largely accountable for the typical characters which most diseases assume; and again, the susceptibility to disease is never constant. It is ever shifting with the stage of development or with the age of the individual. The weak points differ at different periods of life, and at different phases or periods of development.

Another matter of interest and worthy of a few words of inquiry is regarding the value of advice so frequently given by the dentist to his patients respecting diet, advising not infrequently that certain elements or kinds of food, if persisted in, will improve the dental tissues by furnishing the essentials of tooth-structure. Let us for a moment look at the changes which all foods undergo, and see how much encouragement we can gather to sustain this hopeful theory. From the nutritive materials in the blood protoplasm is formed in all the tissues of the body, so that the characteristic elements and products of these tissues must be the result of the constructive changes of this nutritious matter, and in each organ or tissue of the body the protoplasm appears to have special endowments adapted to their specific functions. In illustration, the cells of the glandular organs, such as the salivary glands, the liver, the pancreas, the mammary glands, etc., as well as all the tissues, with their characteristic structural elements, must all be considered as the resulting products of the changing nutritive material. In plant growth the food elements are built up into protoplasm before they are converted into the constituents of plant tissues, such as albumins, starch, fats, etc. So in animal nutrition these same albumins, albuminoids, fats, and carbohydrates must all pass through the preparative stages of blood before they can enter into the composition of the different tissues of the animal body. To trace any specific relation of particular tissues to special food constituents seems out of the question.

In discussing the economics of foods and diets, Dr. Manly Miles says, "If we keep in mind the significant facts *that vital activities direct and determine the transformations of energy and the collocations of matter in plants and animals, in accordance with the nutritive requirements of every organ and tissue*, and that in the higher animals the food supplies of the various tissues that differ so widely in composition and function are derived from the same common pabulum, the blood, which, under the varying conditions of supply and demand, maintains a comparatively uniform composition, the *futility* of assigning to each or any element of the food a specific rôle in the processes of nutrition must be obvious." (Italics are the writer's.)

With the demonstration of one other idea this paper shall close, —to wit, the influence of function or use upon the development of structure; its quality, quantity, and conformation, subjected to modification through this factor.

A well-recognized fact in zoology is the degeneracy which takes place in animals after they become parasitic in their habits. In early life some move about freely with complex organs of locomotion, but when fastened upon or within the animal from which they obtain their subsequent support, organs of special sense as well as of movement are lost. Note also the change which takes place in the eyes of fishes living at great depths in the water, or those swimming after maturity entirely upon one side, as the flat fish, which in early life has its eyes on opposite sides of the head, the submerged eye gradually changing its position until both are looking upward from the same side in response to demands of vision and the animal's protection. If the intelligent oculist or ophthalmologist is consulted, he will tell us that all animals, all savage and uncivilized peoples, all civilized babies, are far-sighted. "The majority of civilized people," says Dr. George M. Gould, "are also hyperopic. Suddenly comes civilization, and within a century, printing, reading, writing, schools, and in-door life are demanding constant use of the eye upon objects within a foot or two, and keeping the ciliary muscle in a state of abnormal continuous tension. The habits and structures of millions of years' formation are in a few years forced to do a work of a very different and straining sort. What is the result? We see, or ought to see, on three-fourths of the civilized portion of the human family some instrument for adapting the vision to this new condition. Nature has been given no time to make the change. One of the unfortunate facts in this eye-strain is that the eye itself frequently does not complain, and is much less conscious of discomfort than other organs, but during all this time the abnormal condition is establishing a group of reflex ocular neuroses, which, though not destroying life, are making it a burden. This wonderful demand which is recognized as being made upon the organs of vision with all its train of pathological results must certainly throw some light upon the manifold abnormal conditions to which the organs of mastication are subjected. Is it possible that one can practise dentistry for twenty, thirty, or forty years without recognizing, first, the great similarity existing between the pathological conditions which he meets in his hundreds of patients; and, noting this fact, must he not be led to the unmistakable conclusion that the important factor behind it all must belong rather to the race, the nation, than the individual? How much less are the dental organs subjected to the influences of civilization than the organs of vision?

The change from uncooked to cooked meats, from fruits and roots to vegetables cooked and the multiplicity of dishes prepared, not only to satisfy man's appetite but to propitiate the palate and stimulate its demands, this must certainly constitute a factor not to be overlooked, and one that must have its influence in modifying those vital activities which direct and determine the transformations of energy and the collocations of matter in accordance with the nutritive requirements of every organ and tissue.

